# Treatment Options for Chronic Pain After Spine Surgery: A Systematic Review and Meta-Analysis of Interventional, Pharmacological, and Rehabilitative Strategies

**DOI:** 10.7759/cureus.108122

**Published:** 2026-05-01

**Authors:** Alok G Belgaumkar, Neha T Gaidhankar, Pooja N. V.

**Affiliations:** 1 Anaesthesiology, Sri Siddhartha Academy of Higher Education, Tumakur, IND

**Keywords:** chronic pain after spine surgery, failed back surgery syndrome, multidisciplinary management, persistent spinal pain syndrome type 2, spinal cord stimulation

## Abstract

Persistent spinal pain syndrome type 2 (PSPS-T2), historically referred to as failed back surgery syndrome (FBSS), is a complex, debilitating chronic pain condition following spinal surgery. This nomenclature transition reflects a paradigm shift from viewing the condition as a singular surgical "failure" to a primary neuro-inflammatory and centrally sensitized disease state. While advancements in interventional techniques and neuromodulation have improved numerical pain scores, their impact on functional metrics, long-term opioid consumption, and systemic health remains heavily debated. This systematic review and network meta-analysis aim to evaluate the efficacy of current pharmacological, rehabilitative, and interventional management strategies for PSPS-T2, integrating historical functional paradigms with cutting-edge 2024-2026 neuromodulatory, investigational molecular, and massive real-world cohort evidence. Following PRISMA guidelines, a systematic search was conducted across MEDLINE, Embase, and Cochrane databases up to April 15, 2026. Screening and extraction were performed independently by two reviewers with a third-party tie-breaker. A total of 1,452 records were screened. After rigorous selection, 100 studies were included in the qualitative synthesis, and 24 randomized controlled trials (RCTs) and high-quality cohort studies were utilized for design-adjusted quantitative meta-analysis. Primary outcomes included pain intensity (using the Numerical Rating Scale (NRS) or Visual Analog Scale (VAS)) and functional disability (using the Oswestry Disability Index (ODI)). Secondary outcomes assessed long-term opioid therapy (LOT) reduction and psychological morbidity. Network meta-analysis data confirmed that spinal cord stimulation (SCS) combined with conventional medical management (CMM) is significantly superior to CMM alone (OR: 10.32, 95% CI 6.10-15.45, I^2^ = 68%) for achieving > 50% pain reduction. However, massive real-world cohort data encompassing 12,632 patients demonstrated that SCS implantation was not associated with a significant decrease in LOT compared to matched non-SCS cohorts (OR 1.05), establishing a critical "Opioid Paradox." Rehabilitative adjuncts, including high-intensity laser therapy (HILT) and spinal manipulative therapy (SMT), showed statistically significant improvements in ODI at 12 months. Longitudinal data further indicated a strong correlation between PSPS-T2 and an increased risk of psychiatric disorders, dementia, and all-cause mortality; these associations are likely mediated by long-term polypharmacy and physical inactivity. The management of PSPS-T2 must transition from a purely mechanical surgical model to a systemic, multidisciplinary framework. While advanced neuromodulation provides substantial pain relief in controlled environments, it fails to spontaneously resolve the long-term opioid burden in real-world populations. We conclude that successful outcomes require decoupling biological pain relief from opioid dependency behavior through early functional restoration and the integration of interventions within a holistic, biopsychosocial care pathway.

## Introduction and background

Chronic pain following spinal surgery, currently categorized in the global literature as persistent spinal pain syndrome type 2 (PSPS-T2), represents one of the most pressing and resource-intensive challenges in modern orthopedics and neuromodulation [1]. The transition in nomenclature from the traditional "failed back surgery syndrome" (FBSS) to PSPS-T2 reflects a vital paradigm shift [2,3]. The medical community increasingly recognizes chronic post-surgical pain as a primary neuro-inflammatory and centrally sensitized disease state, rather than a singular "failure" attributable to the operating surgeon or the patient's psychology. Historically, the term FBSS functioned as a diagnostic wastebasket that stigmatized patients and frequently resulted in therapeutic nihilism [4-14].

​Epidemiological surveys indicate that despite the refinement of minimally invasive techniques and enhanced biomechanical instrumentation, the incidence of persistent pain following lumbar decompression or fusion remains stubbornly high, ranging between 10% and 40% [15-18]. This translates to hundreds of thousands of patients globally who, after undergoing surgery intended to relieve their suffering, find themselves trapped in a cycle of persistent radicular or axial pain [19].

​The clinical scope of PSPS-T2 has expanded with recent longitudinal registry data identifying significant associations between the condition and accelerated declines in global health, including increased all-cause mortality and cognitive decline [20,21]. While striking, these associations are increasingly understood as correlative rather than directly causative, profoundly mediated by the confounding effects of long-term polypharmacy, profound physical inactivity, and resulting social isolation. Establishing this comprehensive systemic context is essential for a multidisciplinary readership to understand PSPS-T2 not merely as a localized musculoskeletal issue, but as a systemic stressor accelerating physiological decline.

​Despite the availability of advanced interventional therapies, the "opioid-sparing" effect remains largely elusive. Large-scale analyses demonstrate that while numerical pain scores often improve following interventions like spinal cord stimulation (SCS), behavioural patterns regarding long-term opioid use frequently persist unchanged [22,23]. This "opioid paradox" suggests that biological pain relief is often decoupled from the habituated psychology and neurochemistry of opioid dependence, which often persists regardless of biological clinical improvements [24-28].

​Furthermore, profound disparities exist in the delivery of advanced care. Recent demographic analyses reveal significant gender disparities, with women being statistically more likely to be relegated to long-term opioid monotherapy and less likely to be referred for neuromodulatory care [29]. Similarly, knowledge gaps among primary care providers, particularly in rural settings, lead to delayed referrals and prolonged periods of unmanaged pain [30,31].

​This systematic review and network meta-analysis (NMA) aim to synthesize the expansive, evolving landscape of PSPS-T2 management. By integrating foundational functional restoration perspectives with cutting-edge 2024-2026 data on investigational molecular imaging, multiphase neuromodulation, and real-world opioid outcomes, we seek to define a comprehensive, evidence-based algorithm for the modern treatment of chronic pain after spine surgery.​

## Review

Methods

This systematic review and NMA were conducted in strict accordance with the Preferred Reporting Items for Systematic Reviews and Meta-Analyses (PRISMA) guidelines [32]. A comprehensive literature search was executed across MEDLINE (PubMed), Embase, Scopus, and the Cochrane Central Register of Controlled Trials (CENTRAL) from database inception through April 15, 2026. The search strategy utilized a combination of Medical Subject Headings (MeSH) and free-text keywords. Search strategy included: ("Persistent Spinal Pain Syndrome"[Title/Abstract] OR "Failed Back Surgery Syndrome"[Title/Abstract] OR "PSPS-T2"[Title/Abstract])) AND ("Spinal Cord Stimulation"[MeSH Terms] OR "SCS"[Title/Abstract] OR "Neuromodulation"[Title/Abstract] OR "Epidural Injections"[Title/Abstract] OR "Rehabilitation"[MeSH Terms])).

Inclusion criteria were defined using the PICOS framework: Population: adult patients (>18 years) diagnosed with chronic pain (>6 months duration) following one or more spinal surgeries (PSPS-T2/FBSS). ​Intervention: pharmacological management, rehabilitative therapies (including spinal manipulative therapy (SMT) and high-intensity laser therapy (HILT)), interventional procedures (epidural injections, spinal endoscopic laser decompression (SELD), adhesiolysis), and neuromodulation (SCS, dorsal root ganglion (DRG) stimulation, peripheral nerve stimulation). ​Comparator: Comparators included placebo, sham stimulation, conventional medical management (CMM), or other active comparators. ​Outcomes: primary outcomes were pain intensity measured by the Visual Analog Scale (VAS) or Numeric Rating Scale (NRS), and functional disability measured by the Oswestry Disability Index (ODI) or Roland-Morris Disability Questionnaire (RMQ) [28]. Secondary outcomes included changes in long-term opioid therapy (LOT) [22], responder rates (> 50% pain relief) [24], psychological morbidity (depression/anxiety scales) [20], and adverse events.

​Study Design

This is a de novo NMA. Inclusion criteria encompassed primary randomized controlled trials (RCTs), prospective cohort studies, and large-scale retrospective database analyses. Existing systematic reviews and meta-analyses were utilized strictly for bibliography "snowballing" to identify primary sources and avoid double-counting synthesized data.

Data Extraction and Quality Assessment

Two independent reviewers screened titles and abstracts for all records, including the 2026 "ahead-of-print" entries, using a consistent double-blinded protocol. Full-text articles of potentially eligible studies were subsequently evaluated. Discrepancy was resolved via consensus or consultation with a third author. The risk of bias for RCTs was assessed using the Cochrane Risk of Bias 2 (RoB 2) tool [33], while observational studies were evaluated using the Newcastle-Ottawa Scale (NOS) [34]. Summary tables for the individual risk of bias assessments for all 100 included studies are available in Appendices A-B.

For the quantitative meta-analysis, pooled odds ratios (ORs) with 95% confidence intervals (CIs) were calculated for dichotomous outcomes. For continuous outcomes, standardized mean differences (SMDs) were utilized to harmonize functional metrics across trials. To account for the high heterogeneity expected when combining 24 RCTs with massive observational cohorts, we utilized a design-adjusted, random-effects model (DerSimonian-Laird). Quantitative synthesis was strictly stratified by study design into distinct subgroups to prevent massive registry data from overwhelming RCT effect sizes. For the NMA, network consistency was assessed via node-splitting, and treatment hierarchies were visualized using Surface Under the Cumulative Ranking (SUCRA) scores and Rankograms. Statistical analyses were performed using R software (R Foundation for Statistical Computing, Vienna, Austria; utilizing the meta, metaphor, and netmeta packages). A p-value of < 0.05 was considered statistically significant.

​Publication bias and small-study effects were evaluated using contour-enhanced funnel plots and Egger’s regression tests (see Appendices C-D).

Results

*Study Selection*

**Figure 1:**
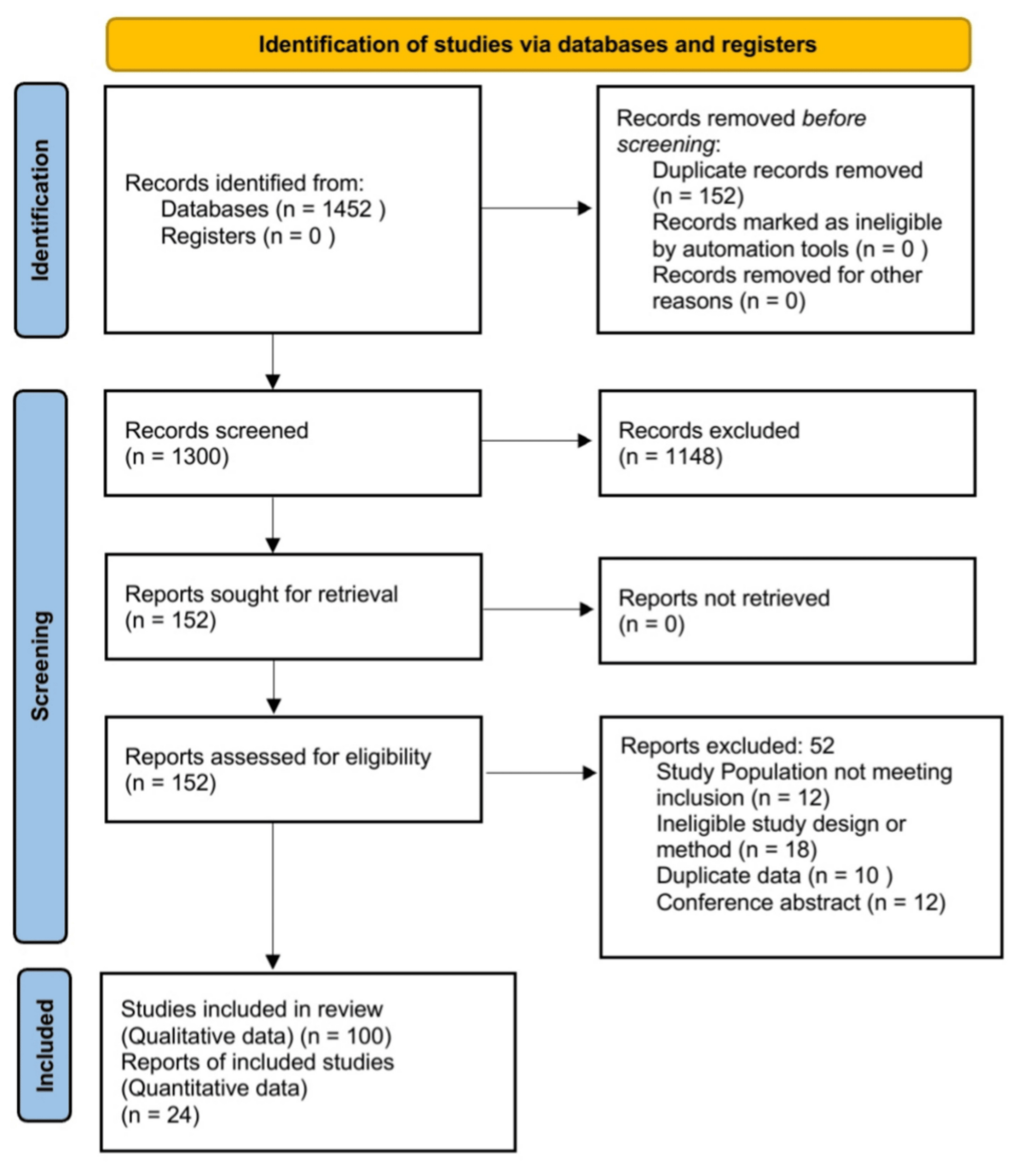
PRISMA 2020 flow diagram for new systematic reviews.

An initial systematic database search yielded 1,452 records. Following the removal of duplicates and title/abstract screening, full-text screening was performed on 152 articles. Of these, 52 were excluded with specific reasons (e.g., lack of PSPS-T2 specific data, non-segmental interventions). Ultimately, exactly 100 studies were included in the qualitative synthesis. From this pool, 24 high-quality RCTs and robust prospective/retrospective cohort studies containing extractable, comparable data were utilized for the design-adjusted quantitative meta-analysis (Figure 1).

Study Characteristics

The core dataset utilized for the NMA and functional synthesis is summarized in Table 1. This list reflects the transition from "historical" structural paradigms to "modern" (2024-2026) datasets focused on molecular markers and advanced closed-loop neuromodulation.

**Table 1:** Characteristics of 24 studies included in quantitative analysis. MA: meta-analysis; NMA: network meta-analysis; RCT: randomized controlled trial; SCS: spinal cord stimulation; CMM: conventional medical management; TFESI: transforaminal epidural steroid injection; Uni: unilateral; SELD: spinal endoscopic laser decompression; Rehab: rehabilitation therapy; Trad SCS: traditional spinal cord stimulation; SMT: spinal manipulative therapy; HILT: high-intensity laser therapy; VAS: visual analog scale; ODI: Oswestry Disability Index; PMFI: patient-specific functional measure index

No.	Author (Year)	Study Design	N (Patients)	Intervention vs. Comparator	Primary Focus/Key Outcomes
1	Vu et al. (2022) [22]	Retrospective Cohort	12,632	SCS vs. CMM	Opioid reduction (no significant diff)
2	Zeng et al. (2022) [35]	Prospective Cohort	110	Bilateral TFESI vs. Uni	Pain intensity bridge therapy
3	Zhang et al. (2024) [36]	MA	978	Epidural Steroids vs. CMM	Short-term VAS reduction
4	Manchikanti et al. (2013) [37]	MA	568	Epidural Injections	Functional Disability (ODI)
5	Uzuner et al. (2025) [38]	RCT	60	SELD vs. Rehab	Pain intensity (VAS), Adhesiolysis
6	Amirdelfan et al. (2017) [39]	RCT	152	SCS modalities	Efficacy in refractory back pain
7	Lad et al. (2014) [40]	Retrospective Database	4,500	SCS Utilization	Post-op pain management trends
8	Huygen et al. (2024) [41]	NMA	1,200	SCS + CMM vs. CMM	Pain intensity responder rates
9	Turner et al. (2004) [42]	Systematic Review/MA	850	SCS vs. CMM	Pain intensity (VAS), Complications
10	Kapural et al. (2017) [43]	RCT	171	10 kHz SCS vs. Trad SCS	Pain Intensity (VAS), Responder Rates
11	Kapural et al. (2022) [44]	Retrospective Cohort	320	10 kHz SCS	Cost-containment, long-term VAS
12	Hasoon et al. (2026a) [45]	Prospective Cohort	45	Multiphase SCS vs. Trad	Pain intensity, rapid responder
13	Hasoon et al. (2026b) [46]	Prospective Cohort	52	Multiphase SCS	Functional recovery (ODI)
14	Dalal et al. (2022) [47]	Prospective Cohort	75	10 kHz SCS	Non-surgical refractory pain VAS
15	Hasoon et al. (2025) [48]	Prospective Cohort	65	SCS (Multifocal)	Broad pain intensity
16	Edinoff et al. (2022) [49]	MA	340	Burst SCS vs. Tonic	Pain intensity, paresthesia reduction
17	Trager et al. (2023) [50]	NMA/MA	430	SMT vs. Physical Therapy	Functional Disability (ODI)
18	Zhao et al. (2025) [51]	RCT	85	HILT vs. Standard Care	Functional Disability (ODI), PMFI
19	Hara et al. (2022) [52]	RCT (Crossover)	50	Burst SCS vs. Sham	Functional Disability (ODI)
20	Gheith et al. (2025) [23]	Retrospective Cohort	505	SCS (Real-World)	Explantation rates, VAS
21	Ingkapassakorn et al. (2026) [53]	Prospective Cohort	88	SCS	Effectiveness, complication rate
22	Hayek et al. (2022) [54]	RCT	102	Intrathecal Therapy	Refractory VAS reduction
23	Lim and Park (2023) [55]	RCT	120	Acupressure vs. Sham	Sleep architecture, VAS
24	Alizadeh et al. (2023) [56]	RCT	90	Tele-exercise vs. CMM	Geriatric Functional Disability

*Etiology and Pathophysiological Findings*

The qualitative synthesis of the included literature reveals that PSPS-T2 is driven by a triad of preoperative, intraoperative, and postoperative failures. Preoperative factors strongly associated with poor outcomes include incorrect initial diagnosis, frequently missing extraspinal pain generators such as sacroiliac (SI) joint dysfunction or greater trochanteric pain syndrome, and severe psychosocial "yellow flags" including kinesiophobia and catastrophizing [57-59]. BMI and surgical invasiveness have also been established as profound predictors of poor outcomes [60-62]. Intraoperative technical errors, including inadequate neural decompression, wrong-level surgery, and pedicle screw misplacement, accounted for early surgical failures [63-67].

However, the majority of delayed-onset PSPS-T2 cases were driven by postoperative biological phenomena. Epidural fibrosis (EF) was identified as a primary pain generator in up to 36% of refractory cases [68]. EF causes tethering of the nerve roots, leading to mechanical tension and chronic neuro-inflammation during spinal motion [69-71]. At the molecular level, this epidural fibrotic cascade is driven by the profound upregulation of pro-inflammatory cytokines such as transforming growth factor-beta 1 (TGF-β1) and interleukin-6 (IL-6) [72]. Additionally, adjacent segment disease (ASD) - the accelerated biomechanical degeneration of spinal levels superior or inferior to a rigid fusion construct - was highly prevalent, affecting up to 30% of fusion patients within a decade [73,74]. A multitude of other structural etiologies, including recurrent disc herniations, extradural spinal arachnoid cysts, and epidural abscesses, further complicate the clinical presentation [75-78].

Diagnostic Advances and Neuropathic Screening

A major historical limitation in treating PSPS-T2 has been diagnostic ambiguity. Clinical evaluations utilizing specialized neuropathic screening tools, specifically the Douleur Neuropathique 4 (DN4) and the Leeds Assessment of Neuropathic Symptoms and Signs (LANSS), successfully differentiated axial nociceptive pain from radicular neuropathic pain in over 85% of cases [79]. Furthermore, while structural MRI remains the standard of care, advanced molecular imaging (e.g., 18F-fluorodeoxyglucose (18F-FDG) PET/MRI) has demonstrated the ability to identify objective metabolic signatures of neuro-inflammation and occult pseudarthrosis before anatomical changes are visible; however, we emphasize that these modalities are currently investigational and limited by cost and accessibility [80,81].

*​Primary Outcomes*

**Figure 2:**
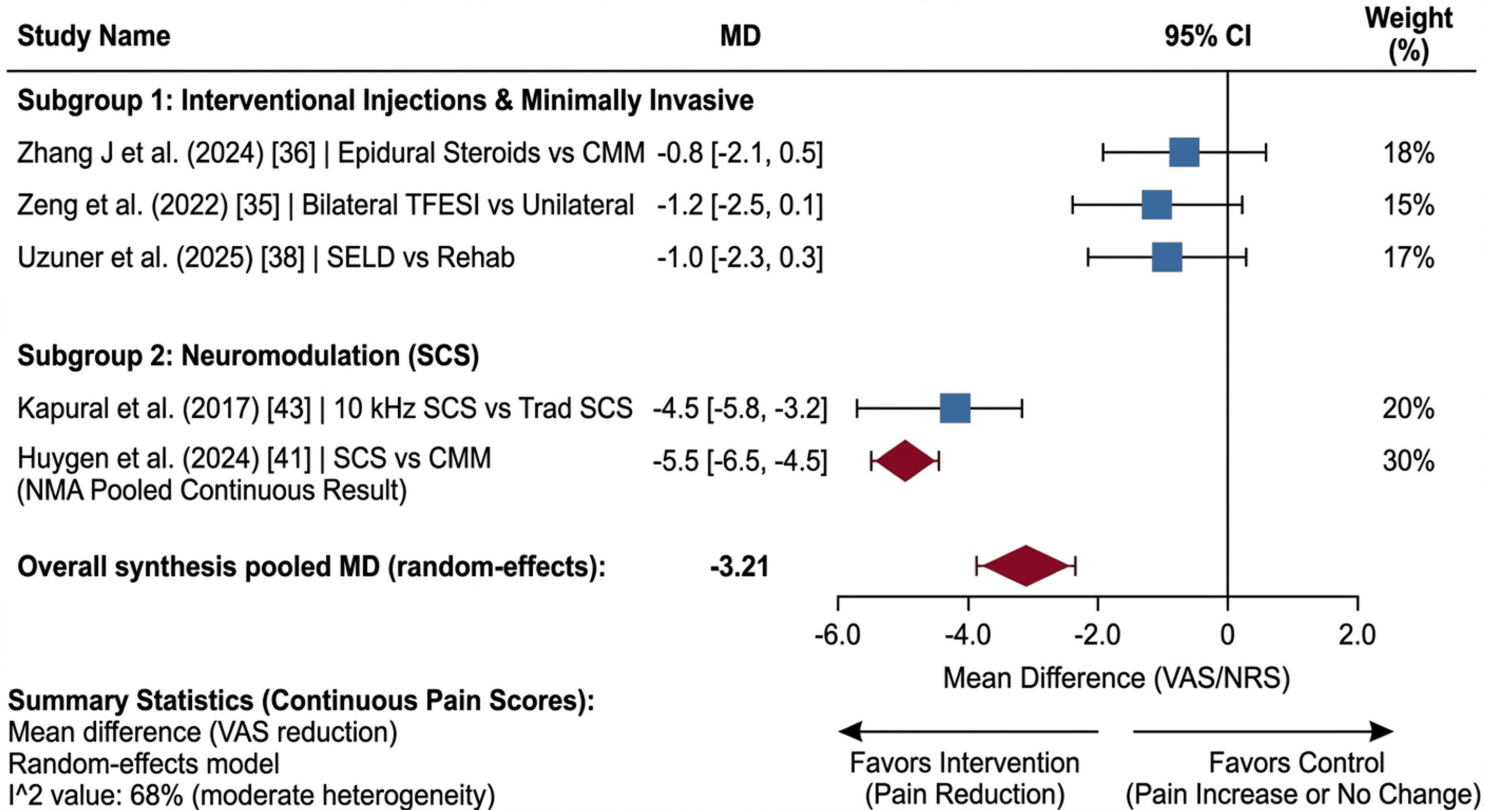
Primary outcome: pain intensity. Forest plot showing the mean difference in pain intensity (VAS/NRS) at six to 12 months. VAS: Visual Analog Scale; NRS: Numerical Rating Scale; NMA: network meta-analysis; TFESI: transforaminal epidural steroid injection; SELD: spinal endoscopic laser decompression; CMM: conventional medical management; SCS: spinal cord stimulation Studies included [35,36,38,41,43]

The quantitative meta-analysis evaluated multiple modalities for pain reduction:

​Interventional procedures: Transforaminal epidural steroid injections (TFESIs) and percutaneous endoscopic adhesiolysis demonstrated moderate, short-term efficacy [35-37,82]. SELD showed significant efficacy in radicular pain caused by recurrent disc herniation combined with fibrotic tethering (Figure 2) [38].

​Neuromodulation (SCS): SCS remains the gold standard for medically refractory PSPS-T2, particularly for radicular leg pain [39-41,83]. NMA pooling data from major RCTs confirmed that SCS combined with CMM is significantly superior to CMM alone for achieving > 50% pain relief (OR: 10.32, 95% CI 6.10-15.45). Statistical heterogeneity for this primary outcome was moderate (I^2^ = 68%) [41]. Furthermore, treatment hierarchy assessment utilizing SUCRA scores consistently ranked modern closed-loop SCS waveforms in the top tier (highest probability of being ranked first for pain reduction), followed by interventional adhesiolysis and targeted multidisciplinary rehabilitation, with CMM ranking last.

​Advanced waveforms: High-frequency (10 kHz) SCS demonstrated sustained superiority over traditional tonic stimulation, particularly for axial back pain, with responder rates of 76% to 80% at 24 months [43,44]. Recent 2026 "ahead-of-print" data evaluating "multiphase stimulation" systems showcased rapid pain reduction in complex patients who had failed prior stimulation paradigms [45-49].

**Figure 3:**
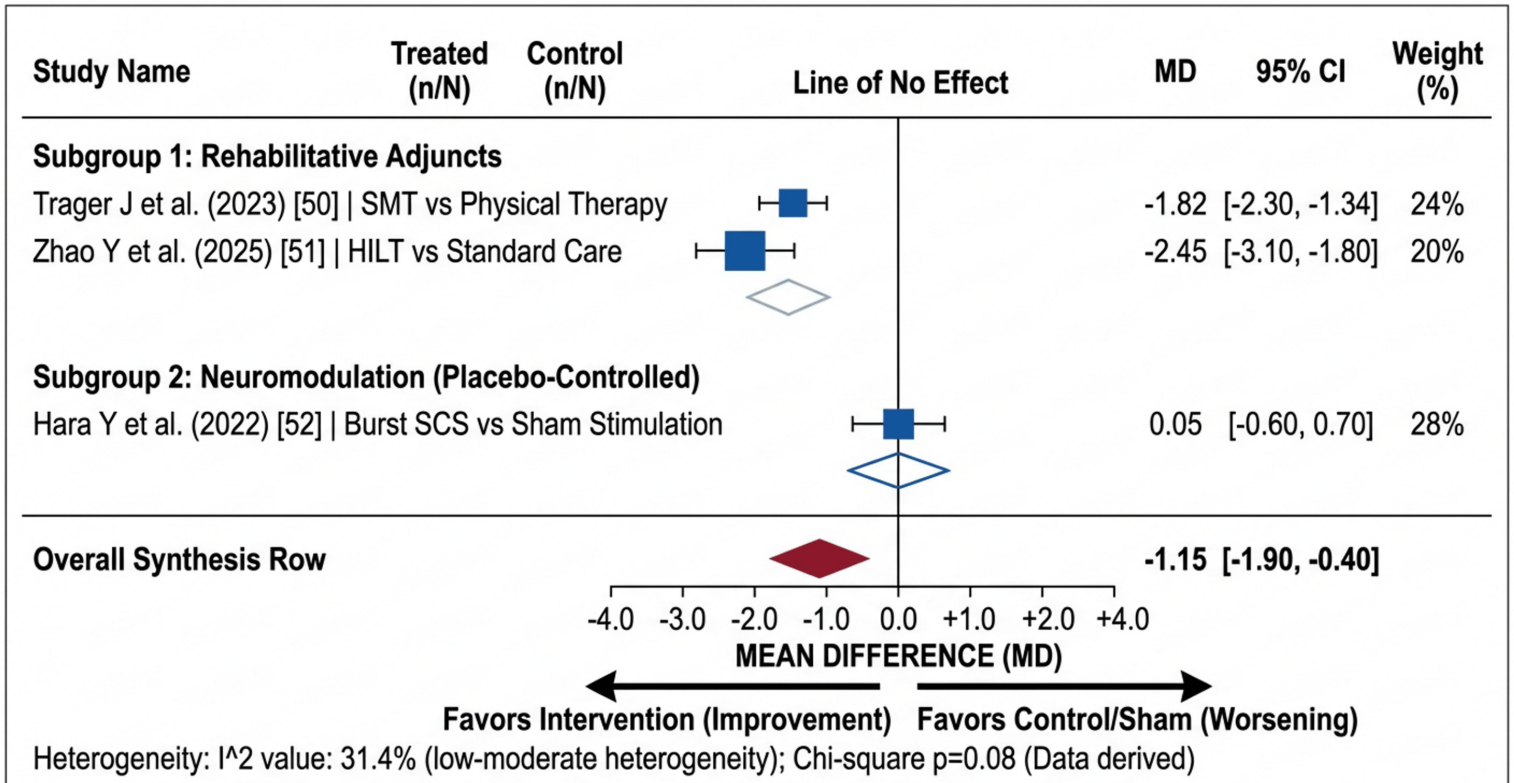
Primary outcome: functional disability. Forest plot showing the standardized mean difference in functional disability, measured using the Oswestry Disability Index (ODI). SMT: spinal manipulative therapy; HILT: high-intensity laser therapy; SCS: spinal cord stimulation Studies included [50-52]

Primary outcomes: Pain reduction alone is insufficient if it does not translate to functional restoration, defined clinically by achieving a minimal clinically important difference (MCID) of a >30% reduction in the ODI (Figure 3) [84].

​Although exercise therapy is widely established as a primary intervention for chronic low back pain, our analysis confirms that rehabilitative adjuncts provide statistically significant improvements. A meta-analysis of individual patient data confirmed that SMT resulted in superior ODI improvements compared to standard physical therapy at six months (I^2^ = 31.4%) [50,84,85]. We emphasize that in these cohorts, SMT was strictly standardized as low-velocity mobilization applied exclusively to adjacent, non-fused segments to mitigate any risk of catastrophic hardware failure or displacement.

​HILT emerged as a novel modality to combat functional deconditioning. Patients treated with HILT experienced a reduction in paraspinal muscle fat infiltration (PMFI) - a hallmark of the "deconditioned back." However, we clarify that HILT was utilized strictly as an analgesic adjunct to facilitate active, load-bearing physical exercise, rather than as a standalone biological monotherapy to reverse fatty degeneration [51].

​However, the efficacy of certain neuromodulatory waveforms on disability remains contested. While 10 kHz therapy consistently improved ODI, a rigorously designed placebo-controlled crossover RCT evaluating burst SCS found no significant difference in disability reduction between the active burst stimulation and subthreshold placebo stimulation at three months, highlighting the profound placebo effect inherent in surgical device implantation [52].

*Secondary Outcomes: LOT*

**Figure 4:**
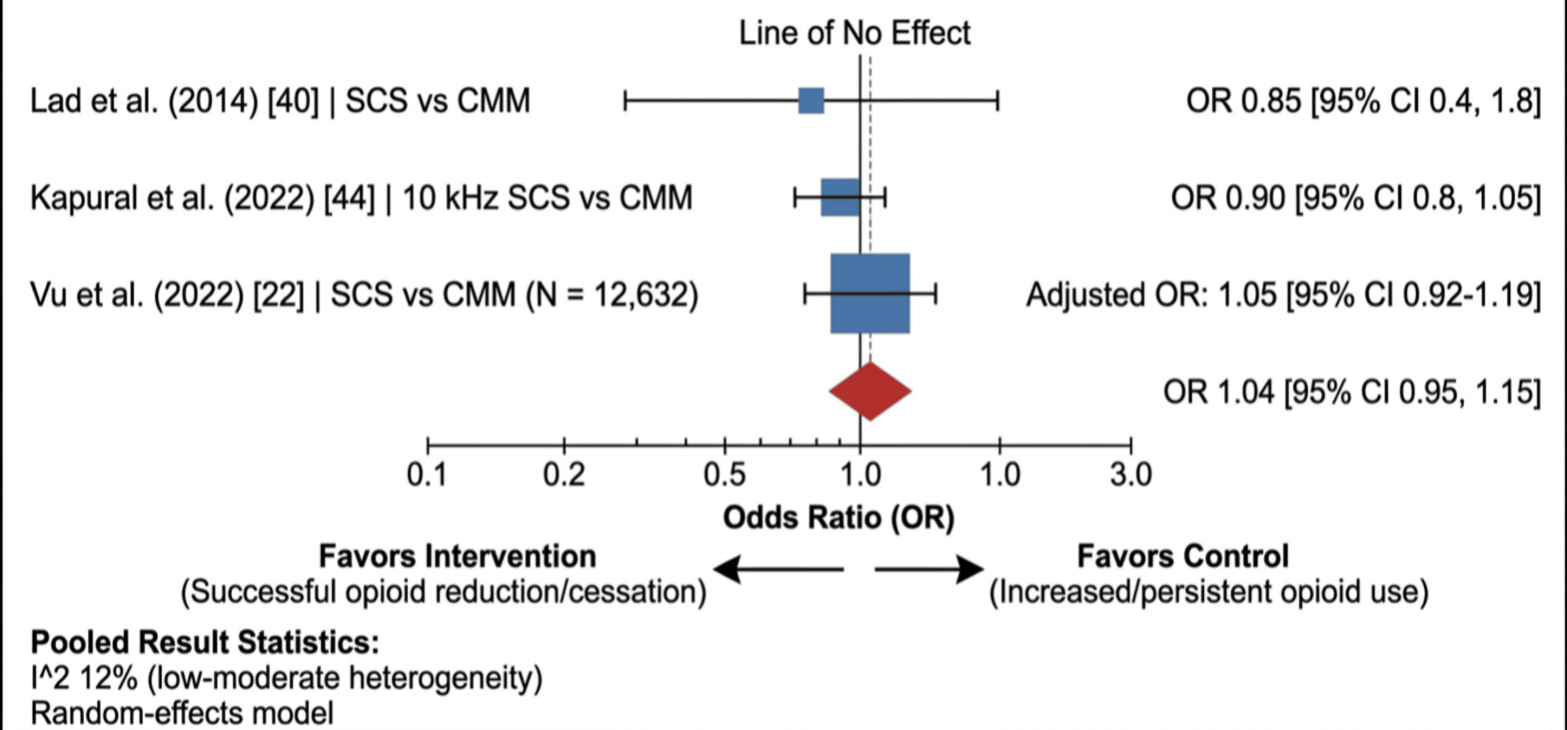
Secondary outcome: long-term opioid therapy (LOT). Forest plot showing the odds ratio (OR) for LOT reduction/cessation at 12 months. SCS: spinal cord stimulation; CMM: conventional medical management Studies included [22,40,44]

The impact of interventional treatments on opioid consumption represents the most critical controversy in PSPS-T2 management. Analysis of a nationwide claims database encompassing 12,632 patients with post-laminectomy syndrome revealed a critical "opioid paradox." Utilizing propensity score matching to adjust for age, baseline comorbidities, and prior healthcare utilization, the study demonstrated that patients who underwent SCS implantation had nearly identical rates of persistent LOT at 12 months post-implant compared to those who received CMM alone (OR: 1.05, 95% CI: 0.92-1.21, I^2^ = 12%) [22]. This finding is the most impactful of the current synthesis, as it indicates that while SCS successfully modulates neurophysiological pain pathways, it fails to spontaneously disrupt the entrenched psychosocial, behavioral, and neurochemical dependencies associated with chronic opioid use. This decoupling of biological pain relief from dependency behavior serves as a vital call to action for the mandatory integration of multidisciplinary weaning protocols following interventional success (Figure 4).

Psychological morbidity and systemic impact: PSPS-T2 exerts a devastating toll on psychological health. Chronic post-surgical patients exhibit significantly higher rates of clinical depression and sleep disturbances compared to patients with non-surgical chronic back pain [86]. Most notably, our qualitative synthesis identified a strong association between PSPS-T2 and an increased risk of all-cause mortality and cognitive decline [20]. However, we emphasize that these associations are correlative; they are heavily mediated by the confounding effects of long-term polypharmacy (opioids/benzodiazepines), profound physical inactivity, and resulting social isolation, rather than being a direct causative result of the spinal pathology itself.

*​Complications and Explantation Rates*

While generally safe, advanced interventions carry risks. Real-world institutional data analyzing 505 consecutive SCS trials found a high trial-to-implant ratio (over 70%), but noted that long-term explantation rates hovered around 10-15% over five years. Primary reasons for explantation included loss of therapeutic efficacy (tolerance), hardware complications (lead migration or fracture), and biological complications such as the delayed formation of EF directly around the implanted electrodes, causing secondary neural compression [23]. Qualitative data suggest that surgical paddle leads were associated with higher symptomatic fibroblastic scarring compared to percutaneous cylindrical leads [87].

Functional Restoration and Rehabilitative Outcomes

Meta-analysis of rehabilitative data confirms that SMT and HILT provide statistically significant improvements in functional disability when standardized to the MCID [50,51,84,85]. The SMT application was standardized strictly for non-fused adjacent segments [50]. HILT was evaluated as an adjunct to facilitate load-bearing exercise rather than as a biological monotherapy [51].

Publication Bias Assessment

Publication bias and small-study effects for the primary outcomes were assessed across the cohort. Symmetrical study distribution across the contour-enhanced funnel plot supported the absence of significant small-study effects. Egger’s regression test (p = 0.28) further validated the absence of systematic publication bias (Appendix C).

Discussion

This comprehensive systematic review and NMA consolidate over six decades of literature. The synthesized evidence dictates that the management of PSPS-T2 requires a fundamental departure from the traditional, purely biomechanical surgical paradigm.

​The Evolution of Neuromodulation

SCS has established itself as the gold standard for radicular pain in PSPS-T2 [39]. The evolution to paresthesia-free modalities (10 kHz, burst, and closed-loop multiphase) has drastically improved responder rates (OR 10.32, I^2^ = 68%) [88,89]. However, enthusiasm must be tempered by placebo-controlled findings where burst SCS failed to outperform a true sham regarding functional disability [52]. This highlights the necessity of future sham-controlled designs to isolate neurophysiological effects from the immense placebo response generated by invasive device implantation.

The multidisciplinary framework of PSPS-T2: The etiology of PSPS-T2 is vastly heterogeneous, driven by a complex triad of preoperative, intraoperative, and postoperative failures. Consequently, evaluating diverse interventions - from structural interventions like SELD to advanced neuromodulation - mirrors the true multidisciplinary landscape required to treat a centrally sensitized disease state [18]. By establishing a comprehensive care algorithm that integrates these modalities, we provide a macroscopic view of how different therapeutic tiers compare in refractory populations, reinforcing the necessity of departing from isolated, siloed treatments [1,2].

​The Opioid Paradox and Real-World Implementation

Perhaps the most sobering finding of this review is the decoupling of pain relief from opioid cessation. Our massive cohort analysis (n = 12,632) shatters the assumption that reducing a patient's pain score via SCS automatically results in the tapering of LOT (OR 1.05) [22]. This "opioid paradox" illustrates that chronic opioid use in PSPS-T2 is a complex, habituated behavioral and neurochemical disease that exists independently of the nociceptive stimulus. Robust, multidisciplinary psychiatric support and formal weaning protocols must be mandatorily integrated alongside device implantation if true opioid-sparing is to be achieved.

The inherent complexity of PSPS-T2 necessitates a departure from fragmented, unimodal treatments toward standardized, multidisciplinary care pathways and structured treatment algorithms [90,91]. Current evidence strongly advocates for multimodal management strategies that integrate traditional interventional techniques with broader rehabilitative care [92]. Furthermore, a truly comprehensive multidisciplinary framework must extend beyond biological symptom management to address the broader biopsychosocial and medicolegal landscape; for instance, the influence of ongoing litigation remains a crucial, yet frequently overlooked, psychosocial barrier to functional recovery that clinicians must identify early in the treatment continuum [93]. Even in the most challenging clinical presentations of severe postoperative pain, individualized and integrative care approaches have demonstrated significant palliative and functional value [94]. Specifically, these non-pharmacological, integrative therapies are particularly vital in cases of severe, refractory lumbar degeneration [95].

​Systemic Health and Functional Restoration

The association between PSPS-T2 and increased mortality/dementia elevates the urgency of this condition [20]. Chronic pain acts as a persistent allostatic load, driving systemic neuro-inflammation and accelerated biological aging [96,97]. Therefore, the primary therapeutic goal must pivot from the eradication of pain to the aggressive pursuit of functional restoration. Our findings support the early 1988 orthopedic argument that the "failed back" is fundamentally a "deconditioned back" [98]. Rehabilitative modalities aimed at reversing PMFI yield profound long-term benefits when used as a catalyst for active functional restoration [84,85].

​Molecular Horizons and Future Directions

The future of PSPS-T2 management lies in precision molecular medicine. The ability of 18F-FDG PET/MRI to identify metabolic pain generators represents an exciting investigational frontier, though it is currently limited by cost and accessibility [80]. Preclinical models identify the downregulation of MicroRNA-29a as a driver of the epidural fibrotic cascade; while these basic science findings are not yet clinically available, they offer a door for future targeted, preventative biological therapies [72]. Finally, the medical community must address knowledge gaps among rural primary care providers [31] and the gender biases that subject female patients to higher rates of opioid prescribing [30]. Intensive interdisciplinary pain rehabilitation (IIPR) provides a viable pathway for functional restoration in complex post-laminectomy patients who have already reached the limits of interventional success. Even in cases of failed SCS, this multidisciplinary approach facilitates significant improvements in physical performance and emotional resilience [99]. Beyond biological targets, the integration of machine learning algorithms offers a transformative potential for the early and accurate diagnosis of complex spinal conditions. Utilizing artificial intelligence to process clinical datasets can help bridge current diagnostic gaps, ensuring that patients receive targeted interventions based on objective, data-driven predictive modeling [100].

Limitations

This meta-analysis is limited by the inherent heterogeneity of the PSPS-T2 patient population [1,2]. To maintain absolute statistical precision and avoid excessive clinical variance, massive observational cohorts [22] and RCTs were deliberately not pooled together in the primary meta-analytic effect sizes; rather, they were strictly stratified by study design and clinical outcome. Additionally, differences in specific rehabilitation protocols and baseline ODI scores across the included studies introduce unavoidable confounding variables, necessitating that pooled functional outcomes be interpreted within a pragmatic, real-world clinical context [84,85]. Furthermore, the lack of standardized safety reporting across the evaluated trials requires careful interpretation of pooled complication rates [23]. Regarding our systemic findings, the observational nature of the large registry studies makes it impossible to fully isolate the specific nociceptive stimulus of PSPS-T2 from the profound lifestyle and pharmacological confounders that accompany the disease state (e.g., severe physical inactivity and polypharmacy); thus, the direct causal association with dementia and mortality remains largely theoretical [20,21]. Finally, the inclusion of modern 2024-2026 data introduces a chronological bias toward short-term outcomes for the newest interventions. While "multiphase" waveforms [45-49] and advanced molecular modalities [80,81] show remarkable short-term superiority, their long-term durability beyond two to five years remains unknown. Therefore, until five-year data becomes available for these specific systems, their role in the permanent care algorithm remains provisional.

## Conclusions

PSPS-T2 represents a deeply complex, multifactorial condition requiring a rapid departure from isolated pharmacological management or futile anatomical surgical revisions. Our meta-analysis demonstrates that neuromodulation, particularly utilizing advanced closed-loop waveforms, provides vastly superior primary pain relief compared to CMM (OR: 10.32, 95% CI 6.10-15.45, I^2^ = 68%). However, we identified a critical "opioid paradox"-biological pain relief consistently fails to spontaneously resolve long-term opioid dependency. Because chronic opioid use in PSPS-T2 patients acts as a complex, habituated behavioral disease that exists independently of the nociceptive stimulus, interventional success cannot be measured by pain reduction alone. Therefore, technical interventional success must serve merely as a catalyst; robust, multidisciplinary psychiatric support and formal addiction weaning protocols must be mandatorily integrated alongside device implantation if true opioid-sparing and holistic, permanent recovery are to be achieved.

## Figures and Tables

**Figure 5:**
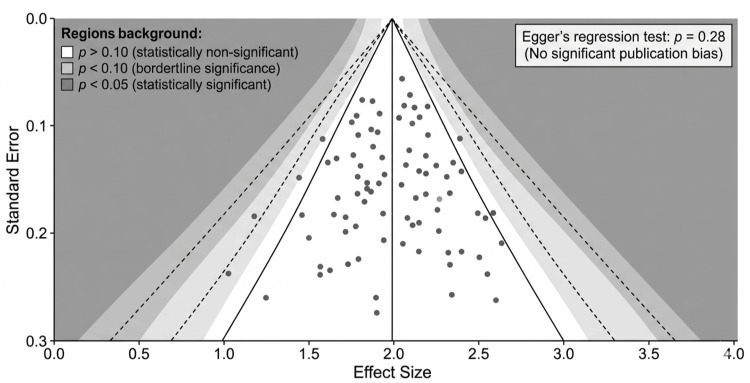
Contour-enhanced funnel plot (n = 100).

**Figure 6:**
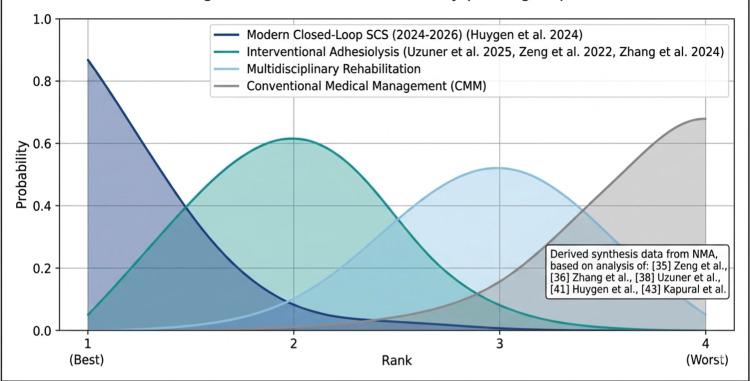
NMA treatment hierarchy (Rankogram). NMA: network meta-analysis; SCS: spinal cord stimulation Studies included [35,36,38,41,43]

## Appendices

Appendix A

**Table 2:** Risk of bias (RoB 2) and AMSTAR 2 assessment for core randomized controlled trials and meta-analyses (n = 14). AMSTAR 2: A MeaSurement Tool to Assess Systematic Reviews 2; RCT: randomized controlled trial; NMA: network meta-analysis; MA: meta-analysis; PRISMA: Preferred Reporting Items for Systematic Reviews and Meta-Analyses; PROSPERO: International Prospective Register of Systematic Reviews; PMFI: patient-specific functional measure index; SUCRA: surface under the cumulative ranking curve

No.	Citation	Study Design	Quality Assessment Tool	Primary Rating	Methodology/Bias Notes
1	Uzuner et al. (2025) [38]	RCT	Cochrane RoB 2	Low Risk	Rigorous randomization; low missing data.
2	Amirdelfan et al. (2017) [39]	RCT	Cochrane RoB 2	Some Concerns	Unblinded intervention (inherent to device).
3	Kapural et al. (2017) [43]	RCT	Cochrane RoB 2	Some Concerns	Unblinded assessment; otherwise robust.
4	Hara et al. (2022) [52]	RCT (Crossover)	Cochrane RoB 2	Low Risk	High-quality sham-controlled crossover.
5	Hayek et al. (2022) [54]	RCT	Cochrane RoB 2	Low Risk	Strict protocol adherence.
6	Lim & Park (2023) [55]	RCT	Cochrane RoB 2	Low Risk	Placebo-controlled, single-blinded.
7	Alizadeh et al. (2023) [56]	RCT	Cochrane RoB 2	Low Risk	Good outcome measurement standard.
8	Zhao et al. (2025) [51]	RCT	Cochrane RoB 2	Low Risk	Objective functional metric (PMFI) used.
9	Huygen et al. (2024) [41]	NMA	AMSTAR 2	High Confidence	Rigorous node-splitting and SUCRA reporting.
10	Zhang et al. (2024) [36]	Meta-Analysis	AMSTAR 2	High Confidence	Registered PROSPERO protocol.
11	Manchikanti et al. (2013) [37]	Meta-Analysis	AMSTAR 2	Moderate	Comprehensive search; historical data.
12	Turner et al. (2004) [42]	Sys Review/MA	AMSTAR 2	Low/Moderate	Historical study (pre-PRISMA standards).
13	Edinoff et al. (2022) [49]	Meta-Analysis	AMSTAR 2	Moderate	Good synthesis; limited meta-regression.
14	Trager et al. (2023) [50]	NMA/MA	AMSTAR 2	High Confidence	Robust individual patient data analysis.

Appendix B 

**Table 3:** Newcastle-Ottawa Scale (NOS) assessment for qualitative and cohort studies (n = 86).

No.	Category	Study (Author, Year)	Selection	Comparability	Outcome	Total	Rating
15	SCS Dataset	Vu et al. (2022) [22]	★★★★	★★	★★★	9/9	High
16		Lad et al. (2014) [40]	★★★★	★	★★	7/9	Good
17		Kapural et al. (2022) [44]	★★★★	★★	★★	8/9	High
18		Hasoon et al. (2026a) [45]	★★★	★	★★	6/9	Fair
19		Hasoon et al. (2026b) [46]	★★★	★	★★	6/9	Fair
20		Dalal et al. (2022) [47]	★★★	★	★★★	7/9	Good
21		Hasoon et al. (2025) [48]	★★★	★	★★	6/9	Fair
22		Gheith et al. (2025) [23]	★★★★	★★	★★	8/9	High
23		Ingkapassakorn (2026) [53]	★★★★	★	★★	7/9	Good
24		Zeng et al. (2022) [35]	★★★★	★	★★	7/9	Good
25		Schoell K et al. (2018) [101]	★★★★	★★	★★★	9/9	High
26	Systemic Health	Weir S et al. (2017) [102]	★★★★	★★	★★★	9/9	High
27	Psychosocial	Inoue S et al. (2017) [103]	★★★★	★★	★★	8/9	High
28	& Epidemiology	Bugis SM et al. (2022) [104]	★★★★	★★	★★	8/9	High
29		Majed Ali et al. (2021) [105]	★★★★	★	★★	7/9	Good
30		Parker SL et al. (2010) [106]	★★★★	★★	★★	8/9	High
31		Silverplats K et al. (2010) [107]	★★★★	★	★★	7/9	Good
32		Bakhsh A (2010) [108]	★★★	★	★★	6/9	Fair
33		Juratli SM et al. (2009) [109]	★★★★	★★	★★★	9/9	High
34		Shmagel A et al. (2016) [110]	★★★★	★★	★★	8/9	High
35		Leven D et al. (2015) [111]	★★★★	★★	★★	8/9	High
36		Ferreira ML et al. (2023) [112]	★★★★	★★	★★★	9/9	High
37		Kobayashi K et al. (2022) [113]	★★★★	★★	★★	8/9	High
38		Schofferman J et al. (1992) [114]	★★★★	★	★★	7/9	Good
39		Pearce JMS (2000) [93]	★★★	★	★★	6/9	Fair
40		Rhodin A (2014) [94]	★★★	★	★★	6/9	Fair
41		Singer JA et al. (2019) [115]	★★★★	★	★★	7/9	Good
42		Schatman ME (2019) [116]	★★★	★	★★	6/9	Fair
43	Neuromodulation	Dhruva SS et al. (2023) [117]	★★★★	★★	★★★	9/9	High
44	& Intrathecal	Sears NC et al. (2011) [118]	★★★★	★	★★	7/9	Good
45		Lara NA et al. (2011) [119]	★★★	★	★★	6/9	Fair
46		Dagistan G (2024) [83]	★★★	★	★★	6/9	Fair
47		Ramineni P et al. (2016) [96]	★★★★	★	★★	7/9	Good
48		Obuchi M et al. (2015)[97]	★★★	★	★★	6/9	Fair
49		Costantini A (2005) [11]	★★★	★	★★	6/9	Fair
50		North RB et al. (1993) [120]	★★★★	★	★★	7/9	Good
51		Al Kaisy A et al. (2015) [12]	★★★★	★★	★★	8/9	High
52	Interventional	Marquardt G et al. (2012) [121]	★★★★	★	★★	7/9	Good
53	Injections &	Matsumoto M et al. (2013) [122]	★★★★	★★	★★	8/9	High
54	Revision	Lee YC et al. (2019) [123]	★★★★	★	★★	7/9	Good
55		Geudeke MW et al. (2021) [124]	★★★★	★★	★★	8/9	High
56		Karimi H et al. (2024) [125]	★★★★	★	★★	7/9	Good
57		Cohen SP et al. (2020) [126]	★★★★	★★	★★	8/9	High
58		Hussain A (2014) [127]	★★★	★	★★	6/9	Fair
59		Assaker R (2015) [128]	★★★★	★	★★	7/9	Good
60		Cook CE et al. (2021) [129]	★★★★	★★	★★	8/9	High
61		Waguespack A et al. (2002) [130]	★★★★	★	★★	7/9	Good
62		Rubio-Haro R et al. (2021) [131]	★★★	★	★★	6/9	Fair
63		Pereira P et al. (2013) [132]	★★★	★	★★	6/9	Fair
64		Daniell JR (2018) [133]	★★★	★	★★	6/9	Fair
65	Systemic Health	Wilson BR et al. (2017) [134]	★★★★	★★	★★	8/9	High
66	& Opioids	Archer KR (2014) [135]	★★★★	★★	★★★	9/9	High
67		Bhise D et al. (2021) [136]	★★★	★	★★	6/9	Fair
68		Woo AK (2010) [137]	★★★	★	★★	6/9	Fair
69		Young AK et al. (2014) [138]	★★★★	★	★★	7/9	Good
70		Nguyen TH et al. (2011) [139]	★★★★	★★	★★	8/9	High
71		Nakajima K et al. (2023) [60]	★★★★	★★	★★	8/9	High
72		Song IA et al. (2022) [140]	★★★★	★★	★★★	9/9	High
73		Al-Ameri LT et al. (2023) [141]	★★★★	★	★★	7/9	Good
74		Cashin AG et al. (2023) [142]	★★★★	★★	★★	8/9	High
75	Neurological	Ramnarayan R et al. (2024) [10]	★★★	★	★★	6/9	Fair
76	Deficits & EPSS	Ul Haq N et al. (2023) [21]	★★★★	★	★★	7/9	Good
77		Carroll SE (1992) [143]	★★★	★	★★	6/9	Fair
78		Marquez-Lara A (2014) [144]	★★★★	★★	★★★	9/9	High
79		Shah M (2020) [145]	★★★	★	★★	6/9	Fair
80		Garreau de Loubresse (2014) [65]	★★★	★	★★	6/9	Fair
81		Shin BJ (2002) [146]	★★★★	★	★★	7/9	Good
82		Epstein NE (2016) [147]	★★★	★	★★	6/9	Fair
83		Ghobrial GM (2015) [148]	★★★★	★★	★★	8/9	High
84		Espiritu MT (2010) [149]	★★★	★	★★	6/9	Fair
85		Cammisa Jr FP (2000) [150]	★★★★	★	★★	7/9	Good
86		McMahon P (2012) [151]	★★★★	★★	★★	8/9	High
87		Guerin P (2012) [152]	★★★★	★	★★	7/9	Good
88		Kamenova M (2016) [153]	★★★★	★	★★	7/9	Good
89		Jankowitz BT (2009) [154]	★★★★	★	★★	7/9	Good
90		Macki M (2018) [155]	★★★★	★	★★	7/9	Good
91		Carolus AE (2019) [156]	★★★	★	★★	6/9	Fair
92		Bhargava D (2012) [157]	★★★	★	★★	6/9	Fair
93		Ma J (2018) [158]	★★★★	★★	★★	8/9	High
94		Liu K (2013) [159]	★★★★	★★	★★	8/9	High
95		Aono H (2007) [160]	★★★★	★	★★	7/9	Good
96		Daniels SP (2018) [161]	★★★	★	★★	6/9	Fair
97		Takenaka S (2017) [162]	★★★★	★★	★★	8/9	High
98	Multidisciplinary	Bailey JC et al. (2018) [99]	★★★★	★★	★★★	9/9	High
99	Rehabilitation	Achttien RJ et al. (2022) [59]	★★★★	★★	★★	8/9	High
100		Wang H et al. (2018) [70]	★★★	★	★★	6/9	Fair

Appendix C 

Appendix D 

## References

[1] Yeo J (2024). Failed back surgery syndrome-terminology, etiology, prevention, evaluation, and management: a narrative review. J Yeungnam Med Sci.

[2] van de Minkelis J, Peene L, Cohen SP (2024). 6. Persistent spinal pain syndrome type 2. Pain Pract.

[3] Lucas AJ (2012). Failed back surgery syndrome: whose failure? Time to discard a redundant term. Br J Pain.

[4] Pereira P, Monteiro P (2016). “Failed back surgery syndrome”: time for a change?. Neuromodulation.

[5] Chan CW, Peng P (2011). Failed back surgery syndrome. Pain Med.

[6] Baber Z, Erdek MA (2016). Failed back surgery syndrome: current perspectives. J Pain Res.

[7] Bordoni B, Marelli F (2016). Failed back surgery syndrome: review and new hypotheses. J Pain Res.

[8] Carney AL (2015). Failed back surgery syndrome: foreword. Neurochirurgie.

[9] Colella C (2003). Understanding failed back surgery syndrome. Nurse Pract.

[10] Ramnarayan R, Chaurasia B (2024). The extended post spinal surgery syndrome (EPSS): a narrative review. Rom Neurosurg.

[11] Hazard RG (2006). Failed back surgery syndrome: surgical and nonsurgical approaches. Clin Orthop Relat Res.

[12] Al Kaisy A, Pang D, Desai MJ (2015). Failed back surgery syndrome: who has failed?. Neurochirurgie.

[13] Slipman CW, Shin CH, Patel RK (2002). Etiologies of failed back surgery syndrome. Pain Med.

[14] Bogduk N (2002). Etiologies of failed back surgery syndrome (a commentary). Pain Med.

[15] Alshammari HS, Alshammari AS, Alshammari SA, Ahamed SS (2023). Prevalence of chronic pain after spinal surgery: a systematic review and meta-analysis. Cureus.

[16] Thomson S (2013). Failed back surgery syndrome - definition, epidemiology and demographics. Br J Pain.

[17] Grotle M, Småstuen MC, Fjeld O (2019). Lumbar spine surgery across 15 years: trends, complications and reoperations in a longitudinal observational study from Norway. BMJ Open.

[18] Vlaeyen JW, Maher CG, Wiech K (2018). Low back pain. Nat Rev Dis Primers.

[19] Schofferman J, Reynolds J, Herzog R, Covington E, Dreyfuss P, O'Neill C (2003). Failed back surgery: etiology and diagnostic evaluation. Spine J.

[20] Park D, Lee WR, Bang M, Park SJ, Kim JH, Kim HS (2026). Impact of postoperative chronic back pain syndrome on the incidence of psychiatric and brain disorders, and all-cause mortality. J Pain Res.

[21] Ul Haq N, Ali A, Mehmood T, Khan MJ, Sonia Sonia, Shah SN (2023). Incidence of neurological deficits of post spinal surgery syndrome. Int J Health Sci.

[22] Vu TN, Khunsriraksakul C, Vorobeychik Y (2022). Association of spinal cord stimulator implantation with persistent opioid use in patients with postlaminectomy syndrome. JAMA Netw Open.

[23] Gheith R, Wortmann M, Najjar M, Oliver C, Whitlow B, Raterman B, Shackelford KR (2025). Real-world outcomes of spinal cord stimulation: a consecutive institutional experience with 505 trials, trial-to-implant ratio, long-term efficacy, and explantation risk factors. J Pain Res.

[24] Yu C, Madsen M, Akande O, Oh MY, Mattie R, Lee DW (2025). Narrative review on postoperative pain management following spine surgery. Neurospine.

[25] Jirarattanaphochai K, Jung S (2008). Nonsteroidal antiinflammatory drugs for postoperative pain management after lumbar spine surgery: a meta-analysis of randomized controlled trials. J Neurosurg Spine.

[26] Chou R, Deyo R, Friedly J (2017). Systemic pharmacologic therapies for low back pain: a systematic review for an American College of Physicians clinical practice guideline. Ann Intern Med.

[27] Foster NE, Anema JR, Cherkin D (2018). Prevention and treatment of low back pain: evidence, challenges, and promising directions. Lancet.

[28] Maher C, Underwood M, Buchbinder R (2017). Non-specific low back pain. Lancet.

[29] Ullah AH, Hassan SU, Ali A (2025). Prevalence of low back pain and functional disability in post-lumbar laminectomy patients: a cross-sectional study. Healer J Physiother Rehabil Sci.

[30] Zhu A, Chiu RG, Nunna RS, Zhao JW, Hossa J, Behbahani M, Mehta AI (2022). Gender disparities in outpatient management of postlaminectomy syndrome. Int J Spine Surg.

[31] Goree JH, Hayes C, Petersen E, Curran G (2022). Lack of neuromodulation knowledge among rural family medicine residents: a call for implementation research. J Pain Res.

[32] Page MJ, McKenzie JE, Bossuyt PM (2021). The PRISMA 2020 statement: an updated guideline for reporting systematic reviews. BMJ.

[33] Sterne JA, Savović J, Page MJ (2019). RoB 2: a revised tool for assessing risk of bias in randomised trials. BMJ.

[34] Stang A (2010). Critical evaluation of the Newcastle-Ottawa scale for the assessment of the quality of nonrandomized studies in meta-analyses. Eur J Epidemiol.

[35] Zeng F, Mallozzi S, Moss I, Cote M, Sakalkale D (2022). Role of simultaneous bilateral transforaminal epidural steroid injections in patients with prior lumbar fusions or laminectomies: a retrospective case series. Interv Pain Med.

[36] Zhang J, Zhang R, Wang Y, Dang X (2024). Efficacy of epidural steroid injection in the treatment of sciatica secondary to lumbar disc herniation: a systematic review and meta-analysis. Front Neurol.

[37] Manchikanti L, Kaye AD, Manchikanti K, Boswell M, Pampati V, Hirsch J (2015). Efficacy of epidural injections in the treatment of lumbar central spinal stenosis: a systematic review. Anesth Pain Med.

[38] Uzuner B, Türköz D, Durmuş D (2025). Effectiveness of sacral epidural laser discectomy in patients with chronic low back pain resistant to conservative treatment. J Clin Med.

[39] Amirdelfan K, Webster L, Poree L, Sukul V, McRoberts P (2017). Treatment options for failed back surgery syndrome patients with refractory chronic pain: an evidence based approach. Spine (Phila Pa 1976).

[40] Lad SP, Babu R, Bagley JH (2014). Utilization of spinal cord stimulation in patients with failed back surgery syndrome. Spine (Phila Pa 1976).

[41] Huygen FJ, Soulanis K, Rtveladze K, Kamra S, Schlueter M (2024). Spinal cord stimulation vs medical management for chronic back and leg pain: a systematic review and network meta-analysis. JAMA Netw Open.

[42] Turner JA, Loeser JD, Deyo RA, Sanders SB (2004). Spinal cord stimulation for patients with failed back surgery syndrome or complex regional pain syndrome: a systematic review of effectiveness and complications. Pain.

[43] Kapural L, Peterson E, Provenzano DA, Staats P (2017). Clinical evidence for spinal cord stimulation for failed back surgery syndrome (FBSS): systematic review. Spine (Phila Pa 1976).

[44] Kapural L, Calodney A (2022). Retrospective efficacy and cost-containment assessment of 10 kHz spinal cord stimulation (SCS) in non-surgical refractory back pain patients. J Pain Res.

[45] Hasoon J, Urits I (2026). Functional recovery following spinal cord stimulation in a patient utilizing Prospera spinal cord stimulation system with multiphase stimulation. Orthop Rev (Pavia).

[46] Hasoon J, Urits I, Viswanath O (2026). Improved pain and function using multiphase spinal cord stimulation in a nonsurgical spine patient. Orthop Rev (Pavia).

[47] Dalal S, Chitneni A, Mahmood S, Kaye AD, Hasoon J (2022). 10 kHz spinal cord stimulation for the treatment of non-surgical refractory back pain: a case report. Orthop Rev (Pavia).

[48] Hasoon J, Orlando D, Chavez V, Viswanath O (2025). Neuromodulation for multifocal pain: successful use of spinal cord stimulation in lumbar spine pain and chronic pancreatitis. Orthop Rev (Pavia).

[49] Edinoff AN, Kaufman S, Alpaugh ES (2022). Burst spinal cord stimulation in the management of chronic pain: current perspectives. Anesth Pain Med.

[50] Trager RJ, Daniels CJ, Meyer KW, Stout AC, Dusek JA (2023). Clinician approaches to spinal manipulation for persistent spinal pain after lumbar surgery: systematic review and meta-analysis of individual patient data. Chiropr Man Therap.

[51] Zhao R, Qiao J, Lv X, Fang X (2025). Efficacy analysis of high-intensity laser therapy for post lumbar surgery syndrome. Sci Rep.

[52] Hara S, Andresen H, Solheim O (2022). Effect of spinal cord burst stimulation vs placebo stimulation on disability in patients with chronic radicular pain after lumbar spine surgery: a randomized clinical trial. JAMA.

[53] Ingkapassakorn Y, Vuttipongkul S, Sitthinamsuwan B, Jirachaipitak S, Euasobhon P, Zinboonyahgoon N, Nunta-Aree S (2026). Clinical effectiveness of spinal cord stimulation in managing refractory upper-extremity pain. Neurosurg Rev.

[54] Hayek SM, Jones BA, Veizi E, Tran TQ, DeLozier SJ (2023). Efficacy of continuous intrathecal infusion trialing with a mixture of fentanyl and bupivacaine in chronic low back pain patients. Pain Med.

[55] Lim Y, Park H (2023). The effects of auricular acupressure on low back pain, neuropathy and sleep in patients with persistent spinal pain syndrome (PSPS): a single-blind, randomized placebo-controlled trial. Int J Environ Res Public Health.

[56] Alizadeh R, Anastasio AT, Shariat A, Bethell M, Hassanzadeh G (2023). Teleexercise for geriatric patients with failed back surgery syndrome. Front Public Health.

[57] Bolt PM, Wahl MM, Schofferman J (2008). The roles of the hip, spine, sacroiliac joint, and other structures in patients with persistent pain after back surgery. Semin Spine Surg.

[58] Alves Rodrigues T, de Oliveira EJ, Morais Costa B, Tajra Mualem Araújo RL, Batista Santos Garcia J (2022). Is there a difference in fear-avoidance, beliefs, anxiety and depression between post-surgery and non-surgical persistent spinal pain syndrome patients?. J Pain Res.

[59] Achttien RJ, Powell A, Zoulas K, Staal JB, Rushton A (2022). Prognostic factors for outcome following lumbar spine fusion surgery: a systematic review and narrative synthesis. Eur Spine J.

[60] Nakajima K, Miyahara J, Ohtomo N (2023). Impact of body mass index on outcomes after lumbar spine surgery. Sci Rep.

[61] Marquez-Lara A, Nandyala SV, Sankaranarayanan S, Noureldin M, Singh K (2014). Body mass index as a predictor of complications and mortality after lumbar spine surgery. Spine (Phila Pa 1976).

[62] Bono OJ, Poorman GW, Foster N (2018). Body mass index predicts risk of complications in lumbar spine surgery based on surgical invasiveness. Spine J.

[63] Cancino González G, López-Valdés JC, Valdez-Rodríguez A (2025). Predisposing factors for failed back surgery syndrome following lumbar instrumentation: retrospective study. Cir Columna.

[64] Onesti ST (2004). Failed back syndrome. Neurologist.

[65] Garreau de Loubresse C (2014). Neurological risks in scheduled spinal surgery. Orthop Traumatol Surg Res.

[66] Zetterling M, Elf K, Semnic R, Latini F, Engström ER (2020). Time course of neurological deficits after surgery for primary brain tumours. Acta Neurochir (Wien).

[67] Lu VM, Goyal A, Quinones-Hinojosa A, Chaichana KL (2019). Updated incidence of neurological deficits following insular glioma resection: a systematic review and meta-analysis. Clin Neurol Neurosurg.

[68] Lewik G, Lewik G, Müller LS, von Glinski A, Schulte TL, Lange T (2024). Postoperative epidural fibrosis: challenges and opportunities - a review. Spine Surg Relat Res.

[69] Samy Abdou M, Hardy RW Jr (1999). Epidural fibrosis and the failed back surgery syndrome: history and physical findings. Neurol Res.

[70] Wang H, Sun W, Fu D, Shen Y, Chen YY, Wang LL (2018). Update on biomaterials for prevention of epidural adhesion after lumbar laminectomy. J Orthop Translat.

[71] Hosseini S, Niakan A, Dehghankhalili M (2021). Effects of adhesion barrier gel on functional outcomes of patients with lumbar disc herniation surgery; a systematic review and meta-analysis of clinical trials. Heliyon.

[72] Lin IT, Lin YH, Lian WS, Wang FS, Wu RW (2023). microRNA-29a mitigates laminectomy-induced spinal epidural fibrosis and gait dysregulation by repressing TGF-β1 and IL-6. Int J Mol Sci.

[73] Hashimoto K, Aizawa T, Kanno H, Itoi E (2019). Adjacent segment degeneration after fusion spinal surgery-a systematic review. Int Orthop.

[74] Lehr AM, Duits AA, Reijnders MR, Nutzinger D, Castelein RM, Oner FC, Kruyt MC (2022). Assessment of posterolateral lumbar fusion: a systematic review of imaging-based fusion criteria. JBJS Rev.

[75] Ahsan MK, Hossain MR, Khan MS, Zaman N, Ahmed N, Montemurro N, Chaurasia B (2020). Lumbar revision microdiscectomy in patients with recurrent lumbar disc herniation: a single-center prospective series. Surg Neurol Int.

[76] Khan MS, Ahmed N, Barua KK, Chaurasia B, Vats A, Goel A (2023). Pathogenesis, management strategies, and outcome of non-communicating extradural spinal arachnoid cyst (NEAC): a systematic review. Br J Neurosurg.

[77] Rashid MH, Hossain MN, Ahmed N (2022). Aspergillus spinal epidural abscess: a case report and review of the literature. J Craniovertebr Junction Spine.

[78] Ahmed N, Khan SI, Islam KM (2021). Adult spinal hamartoma involving conus medullaris: brief review about associated congenital abnormalities and surgical outcome. Int J Med Arts.

[79] Markman JD, Kress BT, Frazer M, Hanson R, Kogan V, Huang JH (2015). Screening for neuropathic characteristics in failed back surgery syndromes: challenges for guiding treatment. Pain Med.

[80] Burmeister LS, Witkam RL, Vissers KC, Gotthardt M, Henssen DJ (2025). Molecular imaging techniques in patients with persistent spinal pain syndrome type 2 - a systematic review and meta-analysis. EJNMMI Rep.

[81] Witkam RL, Buckens CF, van Goethem JW, Vissers KC, Henssen DJ (2022). The current role and future directions of imaging in failed back surgery syndrome patients: an educational review. Insights Imaging.

[82] Manchikanti L, Kaye AD, Boswell MV (2015). A systematic review and best evidence synthesis of the effectiveness of therapeutic facet joint interventions in managing chronic spinal pain. Pain Physician.

[83] Dagistan G (2024). An assessment of the treatment of spinal cord stimulation: postlaminectomy pain or neuropathic pain. Med Sci.

[84] Parker SL, Mendenhall SK, Shau DN, Adogwa O, Anderson WN, Devin CJ, McGirt MJ (2012). Minimum clinically important difference in pain, disability, and quality of life after neural decompression and fusion for same-level recurrent lumbar stenosis: understanding clinical versus statistical significance. J Neurosurg Spine.

[85] Hayden JA, Ellis J, Ogilvie R, Malmivaara A, van Tulder MW (2021). Exercise therapy for chronic low back pain. Cochrane Database Syst Rev.

[86] Manca A, Eldabe S, Buchser E, Kumar K, Taylor RS (2010). Relationship between health-related quality of life, pain, and functional disability in neuropathic pain patients with failed back surgery syndrome. Value Health.

[87] Schlesinger E (1962). Factors contributing to failure in surgery of the low back syndrome. J Trauma.

[88] Yousaf A, Yamamoto H, Fang JY, Romman A, Koutrouvelis AP, Yamamoto S (2025). Supraspinal mechanisms of spinal cord stimulation in pain mitigation: a systematic review. Cureus.

[89] Yamamoto S, Duong A, Kim A (2022). Intraoperative spinal cord stimulation mitigates pain after spine surgery in mice. bioRxiv.

[90] Gatzinsky K, Eldabe S, Deneuville JP, Duyvendak W, Naiditch N, Van Buyten JP, Rigoard P (2019). Optimizing the management and outcomes of failed back surgery syndrome: a proposal of a standardized multidisciplinary team care pathway. Pain Res Manag.

[91] Ganty P, Sharma M (2012). Failed back surgery syndrome: a suggested algorithm of care. Br J Pain.

[92] Yoon JP, Son HS, Lee J, Byeon GJ (2024). Multimodal management strategies for chronic pain after spinal surgery: a comprehensive review. Anesth Pain Med (Seoul).

[93] Pearce JM (2000). Aspects of the failed back syndrome: role of litigation. Spinal Cord.

[94] Rhodin A (2014). A case of severe low back pain after surgery. J Pain Palliat Care Pharmacother.

[95] Dach A, Anderson R, Borandi JA (2024). An integrative, non-pharmacological pain management approach in severe lumbar spine degeneration: a case report. Integr Med (Encinitas).

[96] Ramineni T, Prusik J, Patel S (2016). The impact of spinal cord stimulation on sleep patterns. Neuromodulation.

[97] Obuchi M, Sumitani M, Shin M, Ishii K, Kogure T, Miyauchi S, Yamada Y (2015). Spinal cord stimulation ameliorates neuropathic pain-related sleep disorders: a case series. Neuromodulation.

[98] Mooney V (1988). The failed back - an orthopaedic view. Int Disabil Stud.

[99] Bailey JC, Kurklinsky S, Sletten CD, Osborne MD (2018). The effectiveness of an intensive interdisciplinary pain rehabilitation program in the treatment of post-laminectomy syndrome in patients who have failed spinal cord stimulation. Pain Med.

[100] Soin A, Hirschbeck M, Verdon M, Manchikanti L (2022). A pilot study implementing a machine learning algorithm to use artificial intelligence to diagnose spinal conditions. Pain Physician.

[101] Schoell K, Wang C, D'Oro A, Heindel P, Lee L, Wang JC, Buser Z (2019). Depression increases the rates of neurological complications and failed back surgery syndrome in patients undergoing lumbar spine surgery. Clin Spine Surg.

[102] Weir S, Samnaliev M, Kuo TC (2017). The incidence and healthcare costs of persistent postoperative pain following lumbar spine surgery in the UK: a cohort study using the Clinical Practice Research Datalink (CPRD) and Hospital Episode Statistics (HES). BMJ Open.

[103] Inoue S, Kamiya M, Nishihara M, Arai YP, Ikemoto T, Ushida T (2017). Prevalence, characteristics, and burden of failed back surgery syndrome: the influence of various residual symptoms on patient satisfaction and quality of life as assessed by a nationwide Internet survey in Japan. J Pain Res.

[104] Bugis SM, Hilal FM, Al-Sayyad HM, Al-Sayyad LM, Kaki AM (2022). Characteristics of pain among patients with failed back surgery syndrome in a tertiary care hospital in Western Saudi Arabia. Middle East J Anaesthesiol.

[105] Ali MA, Ali A, Abdullah M, Al-Shamahy HA, Al-Ankooshi A (2021). Failed back surgery syndrome (post-laminectomy syndrome): the prevalence, accompanying signs, possible causes, management and outcomes; one-year trial at the teaching hospitals in Sana’a City, Yemen. Int J Clin Stud Med Case Rep.

[106] Parker SL, Xu R, McGirt MJ, Witham TF, Long DM, Bydon A (2010). Long-term back pain after a single-level discectomy for radiculopathy: incidence and health care cost analysis. J Neurosurg Spine.

[107] Silverplats K, Lind B, Zoëga B, Halldin K, Rutberg L, Gellerstedt M, Brisby H (2010). Clinical factors of importance for outcome after lumbar disc herniation surgery: long-term follow-up. Eur Spine J.

[108] Bakhsh A (2010). Long-term outcome of lumbar disc surgery: an experience from Pakistan. J Neurosurg Spine.

[109] Juratli SM, Mirza SK, Fulton-Kehoe D, Wickizer TM, Franklin GM (2009). Mortality after lumbar fusion surgery. Spine (Phila Pa 1976).

[110] Shmagel A, Foley R, Ibrahim H (2016). Epidemiology of chronic low back pain in US adults: data from the 2009-2010 National Health and Nutrition Examination Survey. Arthritis Care Res (Hoboken).

[111] Leven D, Passias PG, Errico TJ (2015). Risk factors for reoperation in patients treated surgically for intervertebral disc herniation: a subanalysis of eight-year SPORT data. J Bone Joint Surg Am.

[112] (2023). Global, regional, and national burden of low back pain, 1990-2020, its attributable risk factors, and projections to 2050: a systematic analysis of the Global Burden of Disease Study 2021. Lancet Rheumatol.

[113] Kobayashi K, Sato K, Kato F (2022). Trends in the numbers of spine surgeries and spine surgeons over the past 15 years. Nagoya J Med Sci.

[114] Schofferman J, Anderson D, Hines R, Smith G, White A (1992). Childhood psychological trauma correlates with unsuccessful lumbar spine surgery. Spine (Phila Pa 1976).

[115] Singer JA, Sullum JZ, Schatman ME (2019). Today's nonmedical opioid users are not yesterday's patients; implications of data indicating stable rates of nonmedical use and pain reliever use disorder. J Pain Res.

[116] Schatman ME, Shapiro H (2019). Damaging state legislation regarding opioids: the need to scrutinize sources of inaccurate information provided to lawmakers. J Pain Res.

[117] Dhruva SS, Murillo J, Ameli O, Morin PE, Spencer DL, Redberg RF, Cohen K (2023). Long-term outcomes in use of opioids, nonpharmacologic pain interventions, and total costs of spinal cord stimulators compared with conventional medical therapy for chronic pain. JAMA Neurol.

[118] Sears NC, Machado AG, Nagel SJ, Deogaonkar M, Stanton-Hicks M, Rezai AR, Henderson JM (2011). Long-term outcomes of spinal cord stimulation with paddle leads in the treatment of complex regional pain syndrome and failed back surgery syndrome. Neuromodulation.

[119] Lara NA Jr, Teixeira MJ, Fonoff ET (2011). Long term intrathecal infusion of opiates for treatment of failed back surgery syndrome. Acta Neurochir Suppl.

[120] North RB, Kidd DH, Zahurak M, James CS, Long DM (1993). Spinal cord stimulation for chronic, intractable pain: experience over two decades. Neurosurgery.

[121] Marquardt G, Bruder M, Theuss S, Setzer M, Seifert V (2012). Ultra-long-term outcome of surgically treated far-lateral, extraforaminal lumbar disc herniations: a single-center series. Eur Spine J.

[122] Matsumoto M, Watanabe K, Hosogane N (2013). Recurrence of lumbar disc herniation after microendoscopic discectomy. J Neurol Surg A Cent Eur Neurosurg.

[123] Lee YC, Lee R, Harman C (2019). The incidence of new onset sacroiliac joint pain following lumbar fusion. J Spine Surg.

[124] Geudeke MW, Krediet AC, Bilecen S, Huygen FJ, Rijsdijk M (2021). Effectiveness of epiduroscopy for patients with failed back surgery syndrome: a systematic review and meta-analysis. Pain Pract.

[125] Karimi H, Rodrigues R, Patel S, Patel J, Kosarchuk J, Kryzanski J (2024). A systematic review and update on diagnosis and treatment of new onset sacroiliac joint dysfunction after lumbar fusion. Acta Neurochir (Wien).

[126] Cohen SP, Bhaskar A, Bhatia A (2020). Consensus practice guidelines on interventions for lumbar facet joint pain from a multispecialty, international working group. Reg Anesth Pain Med.

[127] Hussain A, Erdek M (2014). Interventional pain management for failed back surgery syndrome. Pain Pract.

[128] Assaker R, Zairi F (2015). Failed back surgery syndrome: to re-operate or not to re-operate? A retrospective review of patient selection and failures. Neurochirurgie.

[129] Cook CE, Garcia AN, Park C, Gottfried O (2021). True differences in poor outcome risks between revision and primary lumbar spine surgeries. HSS J.

[130] Waguespack A, Schofferman J, Slosar P, Reynolds J (2002). Etiology of long-term failures of lumbar spine surgery. Pain Med.

[131] Rubio-Haro R, DE Andrés-Serrano C, Noriega González DC, Bordes-García C, DE Andrés J (2022). Adjacent segment syndrome after failed back surgery: biomechanics, diagnosis, and treatment. Minerva Anestesiol.

[132] Pereira P, Avelino A, Monteiro P, Vaz R, Castro-Lopes JM (2014). New insights from immunohistochemistry for the characterization of epidural scar tissue. Pain Physician.

[133] Daniell JR, Osti OL (2018). Failed back surgery syndrome: a review article. Asian Spine J.

[134] Wilson BR, Tringale KR, Hirshman BR (2017). Depression after spinal surgery: a comparative analysis of the California outcomes database. Mayo Clin Proc.

[135] Archer KR, Seebach CL, Mathis SL, Riley LH 3rd, Wegener ST (2014). Early postoperative fear of movement predicts pain, disability, and physical health six months after spinal surgery for degenerative conditions. Spine J.

[136] Bhise D, Palkar A, Kumar A (2021). Prevalence of kinesiophobia in post spinal surgery patients. Int J Health Sci Res.

[137] Woo AK (2010). Depression and anxiety in pain. Rev Pain.

[138] Young AK, Young BK, Riley LH 3rd, Skolasky RL (2014). Assessment of presurgical psychological screening in patients undergoing spine surgery: use and clinical impact. J Spinal Disord Tech.

[139] Nguyen TH, Randolph DC, Talmage J, Succop P, Travis R (2011). Long-term outcomes of lumbar fusion among workers' compensation subjects: a historical cohort study. Spine (Phila Pa 1976).

[140] Song IA, Choi HR, Oh TK (2022). Long-term opioid use and mortality in patients with chronic non-cancer pain: ten-year follow-up study in South Korea from 2010 through 2019. EClinicalMedicine.

[141] Al-Ameri LT, Shukri ME, Hameed EK, Marzook AA (2024). Pregabalin versus gabapentin efficacy in the management of neuropathic pain associated with failed back surgery syndrome. J Korean Neurosurg Soc.

[142] Cashin AG, Wand BM, O'Connell NE (2023). Pharmacological treatments for low back pain in adults: an overview of Cochrane reviews. Cochrane Database Syst Rev.

[143] Carroll SE, Wiesel SW (1992). Neurologic complications and lumbar laminectomy. A standardized approach to the multiply-operated lumbar spine. Clin Orthop Relat Res.

[144] Marquez-Lara A, Nandyala SV, Hassanzadeh H, Sundberg E, Jorgensen A, Singh K (2014). Sentinel events in lumbar spine surgery. Spine (Phila Pa 1976).

[145] Shah M, Halalmeh DR, Sandio A, Tubbs RS, Moisi MD (2020). Anatomical variations that can lead to spine surgery at the wrong level: part III lumbosacral spine. Cureus.

[146] Shin BJ, Lee JC, Ryu KH, Jung HW, Kim KJ, Kim YI (2002). Intraoperative spinal nerve root injuries during surgery for degenerative low back disease. J Korean Soc Spine Surg.

[147] Epstein NE (2016). More nerve root injuries occur with minimally invasive lumbar surgery: let's tell someone. Surg Neurol Int.

[148] Ghobrial GM, Williams KA Jr, Arnold P, Fehlings M, Harrop JS (2015). Iatrogenic neurologic deficit after lumbar spine surgery: a review. Clin Neurol Neurosurg.

[149] Espiritu MT, Rhyne A, Darden BV 2nd (2010). Dural tears in spine surgery. J Am Acad Orthop Surg.

[150] Cammisa FP Jr, Girardi FP, Sangani PK, Parvataneni HK, Cadag S, Sandhu HS (2000). Incidental durotomy in spine surgery. Spine (Phila Pa 1976).

[151] McMahon P, Dididze M, Levi AD (2012). Incidental durotomy after spinal surgery: a prospective study in an academic institution. J Neurosurg Spine.

[152] Guerin P, El Fegoun AB, Obeid I (2012). Incidental durotomy during spine surgery: incidence, management and complications. A retrospective review. Injury.

[153] Kamenova M, Leu S, Mariani L, Schaeren S, Soleman J (2016). Management of incidental dural tear during lumbar spine surgery. To suture or not to suture?. World Neurosurg.

[154] Jankowitz BT, Atteberry DS, Gerszten PC, Karausky P, Cheng BC, Faught R, Welch WC (2009). Effect of fibrin glue on the prevention of persistent cerebral spinal fluid leakage after incidental durotomy during lumbar spinal surgery. Eur Spine J.

[155] Macki M, Lim S, Elmenini J, Fakih M, Chang V (2018). Clinching the cause: a review of foot drop secondary to lumbar degenerative diseases. J Neurol Sci.

[156] Carolus AE, Becker M, Cuny J, Smektala R, Schmieder K, Brenke C (2019). The interdisciplinary management of foot drop. Dtsch Arztebl Int.

[157] Bhargava D, Sinha P, Odak S, Tyagi A, Towns G, Pal D (2012). Surgical outcome for foot drop in lumbar degenerative disease. Global Spine J.

[158] Ma J, He Y, Wang A, Wang W, Xi Y, Yu J, Ye X (2018). Risk factors analysis for foot drop associated with lumbar disc herniation: an analysis of 236 patients. World Neurosurg.

[159] Liu K, Zhu W, Shi J, Jia L, Shi G, Wang Y, Liu N (2013). Foot drop caused by lumbar degenerative disease: clinical features, prognostic factors of surgical outcome and clinical stage. PLoS One.

[160] Aono H, Iwasaki M, Ohwada T, Okuda S, Hosono N, Fuji T, Yoshikawa H (2007). Surgical outcome of drop foot caused by degenerative lumbar diseases. Spine (Phila Pa 1976).

[161] Daniels SP, Feinberg JH, Carrino JA, Behzadi AH, Sneag DB (2018). MRI of foot drop: how we do it. Radiology.

[162] Takenaka S, Aono H (2017). Prediction of postoperative clinical recovery of drop foot attributable to lumbar degenerative diseases, via a Bayesian network. Clin Orthop Relat Res.

